# Motor abilities and cognitive performance in Latinos with autosomal dominant Alzheimer's disease

**DOI:** 10.1016/j.tjpad.2024.100010

**Published:** 2025-01-01

**Authors:** Andrew J. Petkus, Anup N. Sonti, Lucy Montoya, Abhay Sagare, John M. Ringman

**Affiliations:** a Department of Neurology, Keck School of Medicine, University of Southern California, Los Angeles, CA, USA; b Zilkha Neurogenetic Institute, Keck School of Medicine, University of Southern California, Los Angeles, CA, USA; c Alzheimer's Disease Research Center at the Keck School of Medicine at the University of Southern California, Los Angeles, CA, USA

**Keywords:** Autosomal dominant Alzheimer's disease, Latino's, Cognition, Motor function

## Abstract

**Background:**

Declining motor abilities might be a noninvasive biomarker for Alzheimer's disease (AD). Studying motor ability and AD progression in younger Latinos with autosomal dominant Alzheimer's disease (ADAD) can provide insights into the interplay between motor ability and cognition in individuals with minimal confounding from age-normative changes and comorbid medical conditions.

**Objectives:**

This study aimed to (1) examine motor abilities as a function of years to dementia diagnosis and (2) examine associations between motor ability and cognitive performance.

**Design:**

This was a cross-sectional observational study.

**Setting:**

The study took place at the University of Southern California.

**Participants:**

39 predominately Latino individuals (mean age 38.6 ± 10 years old) known to carry (carriers; n=25) or be at 50% risk for inheriting ADAD but not carrying the mutation (noncarriers; n=14).

**Measurements:**

Individuals completed the motor and cognitive batteries from the National Institute of Health Toolbox (NIHTB) and the Cognitive Abilities Screening Instrument (CASI). All models included effects for age, education, primary language, and sex.

**Results:**

Compared to noncarriers, ADAD mutation carriers had significantly weaker grip strength at 12 years, worse manual dexterity at 10 years, and slower gait speed seven years before the expected age of dementia diagnosis. Worse motor ability was associated with a more severe cognitive disease stage and worse CASI performance, adjusting for demographic and clinical variables.

**Conclusions:**

The findings support the utility of motor performance, precisely grip strength, manual dexterity, and gait speed as potential biomarkers of preclinical AD.

## 1 Introduction

Alzheimer's disease (AD) is a neurodegenerative disorder with a long preclinical phase spanning years to decades before the emergence of the hallmark cognitive symptoms and functional decline [1]. Recently approved disease-modifying therapies only modestly impact functioning [2], emphasizing the need to identify individuals early in the AD-disease process to target for intervention [3]. Over the following decades, the public health significance of AD in the US will grow as the population ages [4]. Even though the US is also becoming more ethnically diverse with a growing number of English- and Spanish-speaking older Latinos, most of the research on pre-symptomatic AD has been with non-Hispanic White populations. With the prevalence of AD in the Latino population expected to grow as the aging population becomes more diverse [5], there is a vital need to understand pre-symptomatic AD in this group [6].

Although decline in episodic memory are the hallmark symptom of AD [7], there is increasing recognition that motor abilities (e.g., gait speed, fine motor dexterity, grip strength, and balance) are also negatively impacted during the AD continuum [8]. Declining motor abilities may be a noninvasive biomarker of preclinical AD, with declines arising approximately ten years before dementia onset [9]. Understanding associations between motor and cognitive performance may be particularly important in the U.S. Latino population due to the high prevalence of several confounding cardiovascular comorbidities (e.g., type II diabetes, strokes, hypertension, and hyperlipidemia) that negatively impact both motor and cognitive ability [10]. Despite this, few studies have examined the association between motor and cognitive performance in older US Latinos.

Autosomal dominant Alzheimer's disease (ADAD), caused by mutations in the *APP, PSEN1*, and *PSEN2* genes with essentially complete penetrance, is the etiology of about 1% of total AD cases. Though there are many similarities in the pathophysiology and clinical presentation to sporadic AD of late-onset [11], differences exist [12], including more frequent gait problems and motor findings in ADAD [[13], [14], [15]]. Notably, the age of dementia onset can be reliably predicted in individuals with ADAD [16] and tends to occur at a younger age, typically in middle adulthood, compared to sporadic forms. Studying patients with ADAD provides a unique opportunity to understand the progression of motor abilities and associations with cognitive functioning with minimal confounding from vascular comorbidities and other age-related changes.

In this study, we compared motor abilities between ADAD mutation carriers and noncarriers as a function of the estimated years to a dementia diagnosis to evaluate whether motor function is impacted in the years leading up to a dementia diagnosis. Next, we examined associations between motor ability and cognitive performance.

## 2 Method

### 2.1 Study participants

This cross-sectional study included 39 predominantly Latino (90%) individuals known to carry (carriers; n=25) or be noncarriers at 50% risk (n=14) for inheriting pathogenic mutations in the *PSEN1* or *APP* genes. Participants were recruited into a study of genetic subtypes of AD among Latinos at the University of Southern California. Participants underwent a comprehensive assessment protocol which included the cognitive and motor batteries from the National Institute of Health Toolbox (NIHTB), neuropsychological testing, the Clinical Dementia Rating Scale [17], multiple neuroimaging modalities, and genetic testing for the mutation they were known to be at risk for inheriting. An estimated duration (in years) to dementia diagnosis was calculated based on the standard approaches using the median age for persons carrying that mutation [16]. The USC Institutional Review Board approved all procedures, and all participants or their proxy provided signed informed consent.

### 2.2 Measures of motor performance

The NIHTB motor battery is a tablet-based (completed on iPad-air 2) assessment of motor function that has been extensively validated and shown to be reliable across the lifespan [18]. The battery includes five measures assessing strength, endurance, locomotion, balance, and dexterity. A brief description of the five subtests is as follows:

*9-Hole Pegboard Dexterity Test:* This measures the participant's ability to manipulate objects with their hands. This test has a speed and accuracy component. Participants were asked to place and remove nine plastic pegs into a plastic pegboard. The raw scores for this test include the time in seconds each participant takes to complete the task. Participants complete the task separately with their dominant and non-dominant hands. The scores reported here were adjusted for gender, age, ethnicity, and education based on the NIHTB normative data. A higher score represents better performance.

*Grip Strength Test:* The Grip Strength Test measures hand grip strength. Participants were asked to squeeze a dynamometer with maximal effort for three seconds while seated with their elbows bent to 90 degrees. Participants complete the test with both their dominant and non-dominant hands. Raw test scores include the force in pounds of the individual's grip for each hand. Raw scores were then adjusted for gender, age, ethnicity, and education based on the NIHTB normative data. A higher score represents stronger grip strength.

*Standing Balance Test:* The standing balance test measures the individual's ability to orient their body in space, maintain upright posture under different conditions, and move without falling. Participants were asked to assume and maintain up to five poses for 50 seconds each. Participants posed with their eyes open on a solid surface (pose 1). Pose 2 includes posing with their eyes closed on a solid surface. This is followed by posing with their eyes open on a foam surface (pose 3) and eyes closed on a foam surface (pose 4). Finally, participants pose with their eyes open in a tandem stance on a solid surface (pose 5). We utilized the age, gender, ethnicity, and education-adjusted total score, representing the relative overall balance ability.

*Four-Meter Walk Gait Speed Test*: The four-meter walk test consists of having the participant perform two walking trials of four meters at their usual pace. The raw score for this test is the number of meters per second. Normative data is unavailable for this test; therefore, we utilized the raw score (in meters per second).

*Two-Minute Walk Endurance Test:* The Two-minute Walk Endurance Test measures walking endurance. Participants continuously walk a 50-foot course (out and back) for two minutes. The raw score of this test is the distance walked in two minutes. The age, gender, ethnicity, and education corrected scores were utilized, with higher scores representing better endurance.

### 2.3 Measures of cognitive performance

The NIHTB cognitive battery is a tablet-based assessment of cognitive performance that is valid and reliable across the lifespan [19,20]. The battery includes seven subtests to assess processing speed, attention, working memory, language, episodic memory, and executive function. A brief description of the seven subtests is provided in the supplemental methods. To reduce the number of statistical comparisons, we utilized the estimated "stage" of cognitive disease for each participant. The cognitive disease stage represents the underlying latent cumulative process of cognitive decline via identifying group-level deviations from cases (carriers) to comparisons (noncarriers) on performance across all five NIHTB cognitive battery tests in an Event-Based Mixture Model. Our previous work describes estimating and validating these stages [21]. A higher stage represents worse cognitive performance.

*Cognitive Abilities Screening Instrument (CASI):* The CASI is a screening test of global cognitive abilities commonly used in cross-cultural epidemiological studies [22]. It consists of 25 items assessing the domains of attention, concentration, orientation, episodic memory, language, visual construction, semantic fluency, abstraction, and judgment. Total scores range from 0 to 100, with higher scores representing better cognitive performance. The CASI has been translated into Spanish and has acceptable reliability and validity Table 1.

**Table 1:** Sample descriptive statistics and comparisons between mutation carriers and non-carriers.

		Mutation status		
Variable	Overall, N = 39	Carrier, N = 25	Non-carrier, N = 14	p-value*
Chronological age (years)	38.64 (10.53)	39.32 (10.68)	37.43 (10.56)	0.60
Sex, n (%)				0.34
Male, n (%)	21 (54)	15 (60)	6 (43)	
Female, n (%)	18 (46)	10 (40)	8 (57)	
Years of formal education	12.46 (3.69)	11.92 (3.03)	13.43 (4.62)	0.29
Latino, n (%)	35 (90)	23 (92)	12 (86)	0.61
**Clinical variables**				
Estimated years until dementia onset	-5.82 (11.42)	-4.44 (9.89)	-8.29 (13.81)	0.37
Clinical Dementia Rating category, n (%)				**0.003**
0	21 (54)	8 (32)	13 (93)	
0.5	7 (18)	6 (24)	1 (7.1)	
1	7 (18)	7 (28)	0 (0)	
2	3 (7.7)	3 (12)	0 (0)	
3	1 (2.6)	1 (4.0)	0 (0)	
Mutation, n (%)				0.66
A431E *PSEN1*	28 (72)	17 (68)	11 (79)	
Other mutations†	11 (28)	8 (32)	3 (21)	
Cognitive Abilities Screening Instrument	78.25 (26.49)	68.48 (28.75)	95.71 (3.61)	**<0.001**
Cognitive disease stage‡	1.53 (2.16)	2.75 (2.35)	0.14 (.36)	**0.008**
**NIHTB Motor Battery Test performance**				
Walking endurance test (T-score§)	44.09 (10.91)	42.48 (12.27)	46.50 (8.34)	0.260
Gait Speed (meters per second)	4.69 (1.11)	4.91 (1.33)	4.33 (0.48)	0.071
Grooved Pegboard Dominant Hand (T-score§)	46.08 (13.77)	40.64 (13.71)	54.64 (8.82)	**<0.001**
Grip Strength Dominant Hand (T-score§)	43.87 (11.30)	39.96 (10.61)	50.57 (9.37)	**0.003**
Balance Test (T-score§)	44.52 (12.27)	44.50 (13.24)	44.54 (11.32)	>0.999

⁎ Welch Two Sample t-test; Fisher's exact test

† For participant confidentiality reasons all other mutations were collapsed into a “other mutations” classification due to the small number of individuals with *PSEN1,* and *APP* mutations other than the A431E *PSEN1* mutation.

‡ Cognitive disease stage was derived via an event-based model comparing carriers and non-carriers on the NIHTB Cognitive Battery Tests, with higher scores representing worse cognitive performance.

§ T-score represents the age, sex, ethnicity, and education-adjusted performance for the respective test, with a higher score representing better performance.

### 2.4 Statistical analysis

We thoroughly examined all variables' data distribution and correlation structure. Mutation carriers were compared to noncarriers on all NIHTB motor tests, cognitive outcomes, and demographic variables. The five Individuals who had estimated dementia onset in over 20 years were winsorized to 20 years to reduce the undue influence from these outliers. Raw performance on NIHTB motor scores and cognitive outcomes was plotted against the estimated years to dementia onset for carriers and noncarriers.

Multivariable linear regression analyses were constructed to address the first research question of whether there were differences in motor abilities between mutation carriers and noncarriers as a function of the estimated years to a dementia diagnosis. The outcome was performance on the respective motor subtest of the NIHTB. Included in the model was a main effect of mutation carrier status (0=noncarrier; 1=carrier), estimated years until dementia onset (-20 to 16), and the carrier by years until dementia diagnosis interaction. The interaction term represents whether carriers perform significantly worse than noncarriers with increasing proximity to the estimated age of dementia diagnosis. We calculated the difference between estimated performance for carriers and noncarriers each year until the dementia diagnosis to determine how many years before the expected dementia diagnosis mutation carriers performed worse than noncarriers. We constructed a separate model for each motor subtest. All regression models also adjusted for the effects of chronological age, sex, and years of education. Individuals were clustered within families to account for the nonindependence of data points.

We constructed another series of multivariable linear regression models to test the second research question, whether motor performance was associated with cognitive performance. Each model included a main effect of each motor subtest, which quantified the association between each motor test performance and each cognitive outcome. All models included chronological age, sex, years of formal education, estimated years until dementia diagnosis, and mutation status as covariates. A separate set of regression models were constructed for each outcome (cognitive disease stage and CASI). Individuals were clustered within families to account for the nonindependence of individuals within families. All regression models were run with the MPLUS statistical software in the R computing environment with the MPLUS Automation package [23].

## 3 Results

### 3.1 Sample characteristics

Participants (N=39) were, on average, 38.6 ± 10.5 years old, mostly of Latino origin (90%), with English (69%) as their primary language, predominately male (54%), with approximately 12.5 ± 3.7 years of formal education (Table 1). Table 1 presents comparisons between ADAD mutation carriers and noncarriers. Over half of the participants were ADAD mutation carriers (n=25; 64%). Mutation carriers were not significantly different from noncarriers regarding age (p=.60), sex (p=.34), years of education (p=.29), or primary language (p=.52). Most ADAD mutation carriers (n = 17, 68%) had the A431E *PSEN1* mutation or were at 50% risk of inheriting this mutation (79%) with the remaining 8 mutation carriers representing 5 different known pathogenic ADAD mutations. The specific breakdown of mutation carriers by mutation is not presented due to the need to conceal at-risk individuals’ mutation status. Carriers were also not significantly different from noncarriers in estimated years until dementia onset (p=.37). Although most participants had a clinical dementia rating of 0 (54%), a significantly larger proportion of mutation carriers had a CDR of 0.5 (n = 7) or higher (n = 11) compared to noncarriers (p = 0.003). Regarding performance on the NIHTB Motor Battery tests, carriers had significantly worse grip strength (p=.003) and grooved pegboard performance (p<.001) compared to noncarriers. Carriers also had marginally slower gait speed compared to noncarriers (p=.071). Carriers were not significantly different from noncarriers regarding balance ability (p>.99) and walking endurance (p=.26). See Supplemental Fig. 1 for a correlation matrix with scatterplots and distributions of demographic, clinical, cognitive, and motor performance variables.

### 3.2 Motor functioning with estimated dementia onset

**Fig. 1:**
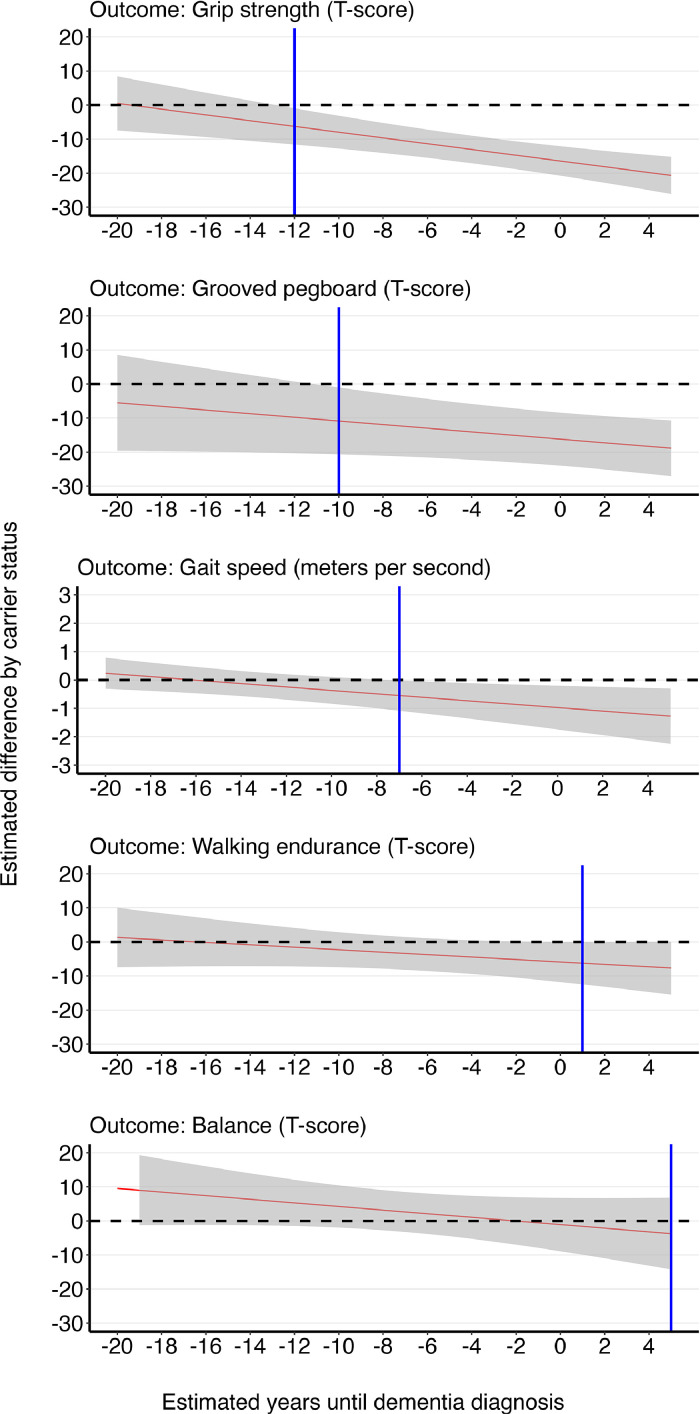
The estimated difference (with 95% confidence interval) in motor performance between mutation carriers and non-carriers as a function of expected years until dementia onset.

The results of the regression analyses examining whether the magnitude of the association between the estimated years until dementia diagnosis and NIHTB Motor battery performance differed by mutation carrier status is presented in Table 2. Significant interactions between carrier status and years to dementia diagnosis were observed on grip strength (p<.001) and gait speed (p=.012), with marginally significant interactions on grooved pegboard (p=.066), balance (p=.146) and walking endurance ability (p=.191). We next probed these interactions by examining the effect of years until dementia diagnosis on motor performance stratified by carrier status. In mutation carriers, advancing years to dementia diagnosis was significantly associated with worse performance on all motor outcomes except for balance ability. In comparison, years to dementia diagnosis were not associated with worse motor performance in nonmutation carriers. We plotted the estimated differences in motor ability between carriers and noncarriers for all five motor tests as a function of years until dementia diagnosis using the effect estimates from the interaction models. As illustrated in Fig. 1, compared to noncarriers, mutation carriers had significantly worse grip strength at 12 years, worse grooved pegboard performance at ten years, and slower gait speed seven years before expected dementia onset. Carriers had significantly worse walking endurance than noncarriers approximately one year after dementia onset, with no significant differences in balance ability (Table 3).

**Table 2:** Results of the multivariable linear regression examining whether mutation status moderated the association between years of dementia on performance on National Institute of Health Toolbox (NIHTB) motor test performance.

	Effect of years to dementia onset on motor performance stratified by carrier status:						Carrier by years to dementia interaction*	
Outcome: NIHTB- Motor Battery Test	Carriers			Noncarriers				
	Est†	95% CI	p	Est†	95% CI	p	Est†	p
Pegboard T-score‡	**-1.28**	**[-1.93; -.63]**	**<0.001**	.35	[-.35; 1.05]	0.327	-0.535	0.066
Grip strength T-score‡	**-.98**	**[-1.41; -.535]**	**<0.001**	.67	[-.02; 1.35]	0.056	**-0.846**	**<0.001**
Gait speed meter/second	**.09**	**[0.01; 0.17]**	**0.030**	-.01	[-0.08; 0.07]	0.852	**1.26**	**0.012**
Walking endurance T-score‡	**-1.02**	**[-1.68; -.35]**	**0.003**	-.78	[-1.70; 0.14]	0.097	-0.359	0.191
Balance T-score‡	-.58	[-1.30; .14]	0.114	.87	[-.12; 1.86]	0.083	-0.531	0.146

Bolded terms denote p<0.05

95% CI = 95% Confidence Interval of parameter estimate

⁎ The carrier by years to dementia continuous interaction term from the full multivariable linear regression moderation model.

† Estimate from the multivariable linear regression adjusting for chronological age, sex, years until expected dementia onset, and mutation status.

‡ T-score represents the age, gender, ethnicity, and education-adjusted performance for the respective test. A higher score represents better performance

**Table 3:** The associations of each National Institute of Health Toolbox (NIHTB) Motor Battery Test performance on cognitive disease stage (Panel A) or Cognitive Abilities Screening Instrument (CASI) performance (Panel B).

Panel A: Effect of NIHTB Motor test on cognitive disease stage			
Predictor: NIHTB Motor Battery Test	Est*	95% Confidence Interval	p
Pegboard (T-score)†	**-.082**	**[-.114; -.050]**	**<0.001**
Grip strength (T-score)†	**-.099**	**[-.164; -.033]**	**<0.001**
Gait speed (meters per second)	**.151**	**[.043; .258]**	**0.006**
Walking endurance (T-Score)†	**-.056**	**[-.106; -.006]**	**0.030**
Balance (T-score) †	-.022	[-.437; 3.522]	0.429

Panel B: Effect of NIHTB Motor Battery test on CASI performance.			
Predictor: NIHTB Motor Battery Test	Est^A^	95% Confidence Interval	p

Pegboard (T-score)†	**.913**	**[.324; 1.502]**	**0.002**
Grip strength (T-score)†	**1.458**	**[.941; 1.975]**	**<0.001**
Gait speed (meters per second)	**-.707**	**[-.964; -.449]**	**<0.001**
Walking endurance (T-score)†	**1.377**	**[.816; 1.938]**	**<0.001**
Balance (T-score)†	.661	[-.005; 1.326]	0.052

Bolded terms denote p<0.05

⁎ Estimate from the multivariable linear regression adjusting for chronological age, sex, years until expected dementia onset, and mutation status.

† T-score represents the age, gender, ethnicity, and education-adjusted performance for the respective test. A higher score represents better performance

### 3.3 Associations between motor and cognitive performance

We next examined the associations between NIHTB Motor Battery test performance with estimates of the overall severity of cognitive disease and CASI performance. Worse performance, per T-score unit, on the grooved pegboard test (p<.001), grip strength (p<.001), and walking endurance tests (p=.006) were associated with a higher estimate of cognitive disease stage. Slower gait speed, per one meter per second, was also significantly associated with a more severe estimated cognitive disease stage. Worse pegboard, grip strength, and walking endurance test performance were also associated with significantly worse performance on the CASI. Slower gait speed, per one meter per second, was also associated with significantly worse performance on the CASI. Motor tests were not significantly associated with balance performance. All estimates were adjusted for sex, chronological age, years until expected dementia onset, and mutation status.

## 4 Discussion

In this cross-sectional study, we examined motor performance, as measured by the NIHTB Motor Battery, as a function of estimated years until dementia onset in mostly middle-aged Latinos at risk for ADAD. Compared to noncarriers, we found that carriers of the ADAD mutation had significantly worse performance on fine-motor dexterity (pegboard performance) and grip strength while having marginally slower gait speed. Furthermore, the differences in motor performance between mutation carriers and noncarriers were of larger magnitude with increasing proximity to the expected onset of dementia. Compared to noncarriers, carriers had significantly worse grip strength approximately 12 years before the anticipated age of dementia diagnosis. Mutation carriers also had significantly worse manual dexterity ten years and slower gait speed seven years before the expected age of dementia diagnosis compared to noncarriers of the mutation. Walking endurance was not impacted until after the expected dementia diagnosis, with carriers performing worse than noncarriers three years after dementia diagnosis. For carriers, motor performance, except for balance, was significantly worse with increasing proximity to the estimated age of dementia diagnosis. In noncarriers, the association between the expected years until dementia diagnosis and motor performance was not significant. The familial comparison group allows us to adjust rigorously for unmeasured shared familial and other additive genetic influences. Poorer performance on all motor tests, except balance, was associated with worse cognitive ability as estimated by the total of individuals' performance on the NIHTB Cognitive battery and a commonly used screening measure. The current study is the first, to our knowledge, to report that motor abilities begin to be impacted in Latinos with ADAD more than ten years before the expected diagnosis. Furthermore, even in this relatively young sample without a high burden of comorbid conditions, worse motor ability was associated with worse cognitive ability.

Our findings that motor abilities are negatively impacted across the process of ADAD are consistent with previous literature on individuals with late-onset sporadic AD and normal cognitive aging [[24], [25], [26]]. Meta-analytic studies report that weaker grip strength was associated with a 1.45 increased risk of AD, while slow gait speed was associated with a 1.03 increased risk of AD dementia [27,28]. Nearly all previous studies have examined these associations in older adults and their relation to late-onset sporadic Alzheimer's disease. Motor abilities decline throughout older age, even after adjusting for brain pathologies [26]. The presence of confounding variables that may contribute to declines in motor ability and cognitive performance that are more common in older adulthood, such as obesity, vascular disease, and musculoskeletal disorders, which are likely contributing to declines in motor ability and AD neuropathology is an important limitation of the previous work. The current study provides novel data addressing these limitations by demonstrating that motor abilities are negatively impacted during the preclinical phase of early-onset ADAD. The confounding variables that are limitations in studies with late-onset AD are likely not impacting our results due to the relatively young sample (mean age 38 years old) where these confounders are less prevalent. Lastly, the current study adds to the literature by demonstrating that motor functions are negatively impacted in the preclinical phase of ADAD in a predominantly ethnically diverse sample of Latinos.

Findings from our study support the likelihood that neuropathological processes occurring over the ADAD continuum negatively impact motor and cognitive abilities [26,[29], [30], [31]]. The accumulation of AD neuropathological factors may commonly impact motor and cognitive ability. Cross-sectional studies in AD of late-onset report associations between cerebral amyloid-β (Aβ) deposition and worse motor abilities, including slower gait speed and weaker grip strength [31,32]. Epidemiological studies with neuropathological factors measured at autopsy report that individuals with more neurofibrillary tangles, and transactive response DNA binding protein-43 (TDP-43) experienced significant declines in grip strength but not gait speed compared to individuals with less neurofibrillary pathology [26,33] and less TDP-43 accumulation [33]. Our finding is consistent with this work as mutation carriers had significantly worse grip strength than noncarriers approximately 12 years before expected dementia onset, suggesting the impact of AD neuropathological factors. We have observed abnormalities in white matter indexed using diffusion tensor imaging in association with spastic paraparesis among carriers of the A431E *PSEN1* mutation [34], potentially indicating the involvement of processes to some extent unrelated to Aβ or neurofibrillary pathology. In addition to these neuropathological factors, common brain structures impacted over the ADAD continuum may play an essential role in cognition and motor abilities. The medial temporal lobe is affected early in the ADAD neurodegenerative process and is implicated in gait speed [35], grip strength [36] and episodic memory [37]. The prefrontal cortex and motor cortex are other brain structures negatively impacted by ADAD and are also involved in motor [38,39] and cognitive abilities, including executive function, episodic memory, and attention. Future research is needed to elucidate mechanisms, including neuropathological burden (e.g., neurofibrillary TAU tangles, TDP-43, and neurofilament light) and cortical brain atrophy that may be contributing to declines in both motor and cognitive abilities

The current study also illustrates the promise of new approaches to understanding the associations between motor and cognitive performance in individuals at risk for ADAD. First, to the best of our knowledge, this was the first study to utilize the increasingly employed tablet-based NIHTB battery to measure motor and cognitive performance. Demonstrating these associations with the tablet-based, relatively brief NIHTB batteries is vital as they support future studies to utilize these tools, which are tablet-based, relatively short, with minimal patient burden compared to comprehensive neuropsychological evaluation. Second, we utilized two cognitive outcomes: a commonly used screening measure of global cognitive abilities (the CDR) and an EBM-derived estimate of each individual's stage of cognitive disease that was derived using the entirety of the NIHTB cognitive battery. The robust associations between motor performance and this estimate of the cognitive stage further demonstrate the potential usefulness of the EBM approach to staging cognitive performance.

Our findings have important clinical implications for practitioners. First, motor deficits, specifically in grip strength, manual dexterity, and gait speed, may be relatively quickly assessed biomarkers of preclinical AD. Routine tests of these motor abilities by practitioners may improve the identification of individuals who are at increased risk for AD to target for primary or secondary prevention efforts. Secondly, our results raise the possibility that interventions designed to improve motor function (e.g., physical therapy, physical exercise) may also positively impact slowing cognitive decline. The results suggest that cognitive remediation interventions may positively impact motor ability. Finally, our findings highlight the importance of addressing cognitive and motor abilities in people at risk for AD.

We recognize several limitations of our study. First, our sample was not very large, and future research needs to replicate these findings with a larger sample size. Additionally, 17 carriers had a CDR of 0.50 or higher. Ideally, all participants would be at the preclinical stage of a CDR of zero. Unfortunately, we were unable to conduct sensitivity analyses including only individuals with a CDR of zero due to limited sample size (n=8 carriers with a CDR of zero). Second, despite the ability to reliably estimate the estimated time until a dementia diagnosis and including familial controls, the data for this study was cross-sectional. As a result, we cannot make inferences about changes in motor ability or cognitive ability over time or inferences regarding causality. Third, most of the sample were carriers or at risk of carrying the A431E [[40], [41], [42]], and two were overtly symptomatic carriers of the F388S *PSEN1* mutation [43]. As can be seen with other *PSEN1* mutations, carriers of these mutations often present with spastic paraparesis in the lower extremities, which progresses over time to affect the upper extremities in the advanced stages of the disease. Although our findings suggest that motor abilities in the upper extremities (e.g., grip strength and pegboard performance) were initially impacted, whether these findings were driven solely by carriers with these mutations cannot be ascertained with this small sample size. Fourth, although the NIHTB motor battery contains well-validated and commonly used motor tests, the four-meter gait speed test does not include normative data. Finally, we related motor dysfunction to overall cognition. It may be that specific domains (e.g., slowing of cognition) may be better correlated with motor dysfunction than others, and identifying such associations could shed light on a common underlying neuroanatomy and neuropathology.

In conclusion, in this study using cross-sectional data in a predominantly Latino population, we found that carriers of ADAD mutations had significantly worse grip strength, manual dexterity, gait speed, and walking endurance than familial noncarriers at 50 percent risk of inheriting the mutation. Differences between carriers and noncarriers were observed as far out as 12 years before the age of expected dementia onset. We also observed significant adverse associations between motor and cognitive performance regardless of mutation status. The findings support the utility of motor performance, specifically grip strength, manual dexterity, and gait speed, as potential biomarkers of preclinical AD.

## Funding

This study was supported by R01AG062007, U01AG051218, and P30AG066530. The sponsors had no role in the design and conduct of the study, in the collection, analysis, and interpretation of data, in the preparation of the manuscript, or in the review or approval of the manuscript.

## Ethics statement

All participants or their medical proxies provided informed consent before study participation. This research was conducted under approval from the Institutional Review Board at the University of Southern California (IRB number HS-19-00707).

## Contributor statement

AJP conceptualized the study, completed the data analysis and manuscript write-up, and is the guarantor of the work. JR contributed to the study's conceptualization, obtained funding, provided advice to the analysis plan, and contributed to the manuscript write-up. AS contributed to the manuscript write-up and the methodology. AS contributed to the manuscript write-up and methodology. LM contributed to the manuscript write-up and methodology.

## Declaration of competing interest

None declared.

## Appendix Supplementary materials

Image, application 1

Image, image 2
