# Supplementary material for: Motor abilities and cognitive performance in Latinos with autosomal dominant Alzheimer's disease
Source: J Prev Alzheimers Dis. 2025 Jan 1;12(1):100010. doi: 10.1016/j.tjpad.2024.100010 (PMC12184005; doi:10.1016/j.tjpad.2024.100010)
Supplement: Supplementary file 1 [file mmc1.docx]

**Supplemental methods**

**NIH Toolbox Cognitive Battery**

The NIHTB cognitive battery is a tablet-based assessment of cognitive performance that is valid and reliable across the lifespan (Weintraub et al., 2013, 2014). The battery includes seven subtests that are designed to assess the domains of processing speed, attention, working memory, language, episodic memory, and executive function. Participants completed the NIH-TB on an iPad Air 2. A brief description of the seven subtests is as follows:

*Pattern Comparisons Processing Speed:* During this test, participants are presented with two stimuli on the tablet screen. Participants are instructed to indicate as quickly as they can using their dominant hand if the presented stimuli are the same or different. Participant’s score on this test is the number of items correctly answered in 85 seconds.

*Flanker Inhibitory Control and Attention:* The Flanker test measures both attentional ability and the inhibitory control aspect of executive function. Participants are asked to focus on a stimulus, an arrow pointing horizontally while inhibiting attention to other stimuli that are “flanking” the target stimulus. Participants are asked to indicate the direction the arrow is pointing by tapping the corresponding response button on the screen. All participants completed 20 items and the score is based on the speed of response.

*List Sorting Working Memory:* A series of stimuli are presented to the participant on the tablet screen. In the first condition, participants are asked to order the stimuli that were presented on the screen from smallest to largest. During the second condition, participants are presented with a series of pictures of food and animals. Participants are instructed to order the food from smallest to largest followed by the animals from smallest to largest. The participant’s score is the sum of the total items correctly sequenced ranging from 0-26. The List Sorting test assesses working memory.

*Picture Vocabulary:* The Picture Vocabulary test assesses the receptive vocabulary aspect of language. Participants are presented with an audio recording of a word followed by four images presented visually on the screen. Participants are asked to select the image that corresponds with the word that is presented to them. This test is administered in a computerized adaptive format that uses Item Response Theory (IRT) to estimate each participant’s overall ability. The IRT-derived score is known as a theta score and has a mean of zero and a standard deviation of one.

*Oral Reading Recognition:* The Oral Reading Recognition Test assesses language ability. Participants are presented with a sequence of words and are asked to read each word as accurately as possible. IRT is again used to score the Oral Reading Test with a theta score calculated for each participant.

*Picture Sequencing Memory:* The Picture Sequencing Memory Test assesses episodic memory. Participants are presented with images and audio statements of events sequentially. The images are then scrambled in the center of the tablet screen. Participants are instructed to replicate the sequence of events by dragging the images to their appropriate position on the screen. The Picture Sequence Memory Test is an adaptive test with a theta score generated using IRT. Higher scores represent better episodic memory.

*Dimensional Card Sorting:* The Dimensional Card Sorting Test assesses the cognitive flexibility aspect of executive function. Participants are asked to match a series of test pictures to a target pictures. Participants are asked to match the pictures based on colors or shapes. After four trials of matching in a certain fashion the participants are asked to switch to matching the test cards differently. Scoring is based on a combination of accuracy and reaction time.
