# Supplementary figures and images for: Motor abilities and cognitive performance in Latinos with autosomal dominant Alzheimer's disease
Source: J Prev Alzheimers Dis. 2025 Jan 1;12(1):100010. doi: 10.1016/j.tjpad.2024.100010 (PMC12184005; doi:10.1016/j.tjpad.2024.100010)

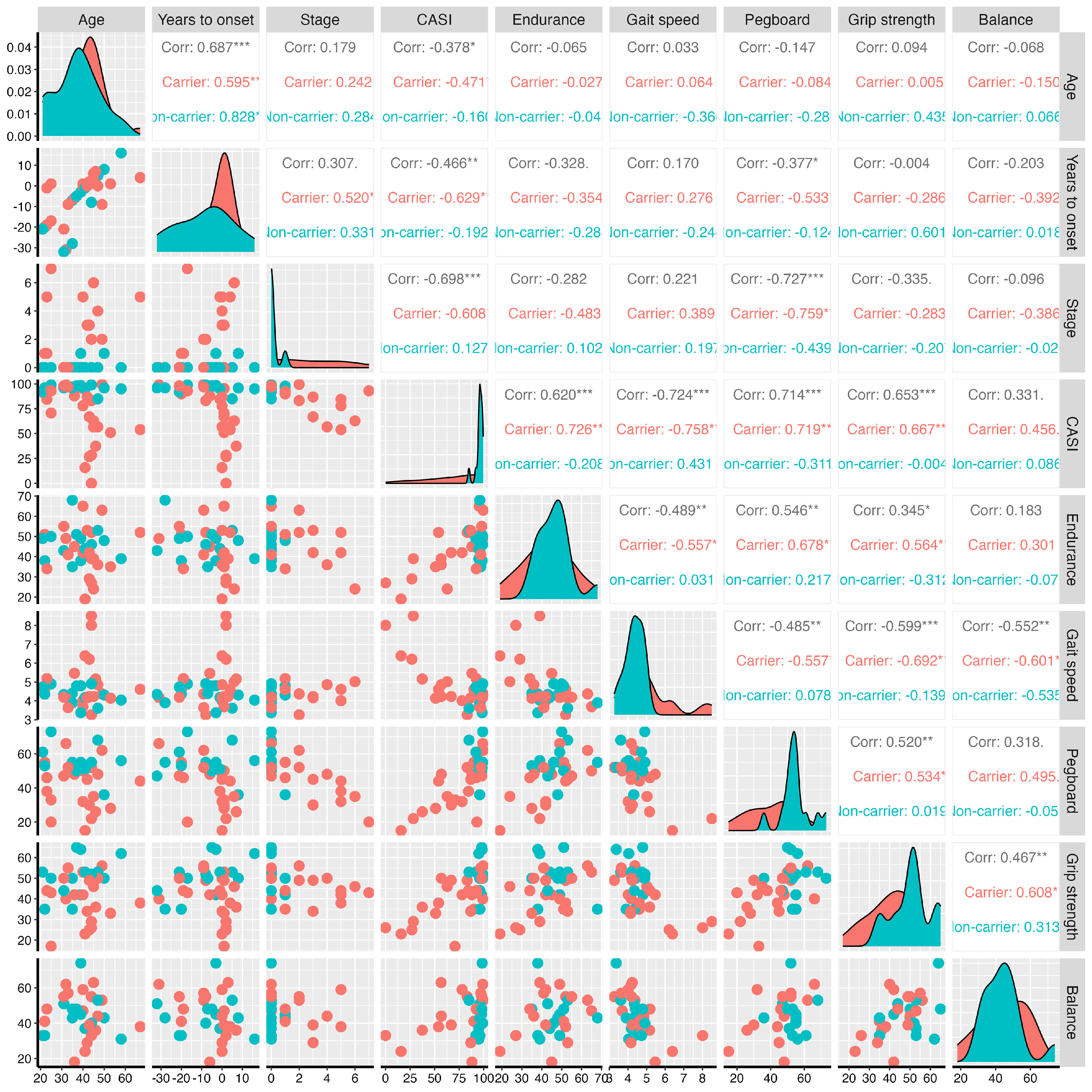

Supplement: Supplementary file 2 [file mmc2.jpg]
